# Adjuvant Effect of Killed *Propionibacterium acnes* on Mouse Peritoneal B-1 Lymphocytes and Their Early Phagocyte Differentiation

**DOI:** 10.1371/journal.pone.0033955

**Published:** 2012-03-20

**Authors:** Juliana Sekeres Mussalem, Carla Cristina Squaiella-Baptistão, Daniela Teixeira, Tatiana Mina Yendo, Felipe Garutti Thies, Ana Flavia Popi, Mario Mariano, Ieda Longo-Maugéri

**Affiliations:** 1 Disciplina de Imunologia, Departamento de Microbiologia, Imunologia e Parasitologia, Universidade Federal de São Paulo – Escola Paulista de Medicina, São Paulo, Brasil; 2 Laboratório de Imunoquímica, Instituto Butantan, São Paulo, Brasil; Aarhus University, Denmark

## Abstract

B-1 lymphocytes are the predominant cells in mouse peritoneal cavity. They express macrophage and lymphocyte markers and are divided into B-1a, B-1b and B-1c subtypes. The role of B-1 cells is not completely clear, but they are responsible for natural IgM production and seem to play a regulatory role. An enriched B-1b cell population can be obtained from non-adherent peritoneal cell cultures, and we have previously demonstrated that these cells undergo differentiation to acquire a mononuclear phagocyte phenotype upon attachment to the substrate *in vitro*. Nevertheless, the B-1 cell response to antigens or adjuvants has been poorly investigated. Because killed *Propionibacterium acnes* exhibits immunomodulatory effects on both macrophages and B-2 lymphocytes, we analyzed whether a killed bacterial suspension or its soluble polysaccharide (PS) could modulate the absolute number of peritoneal B-1 cells in BALB/c mice, the activation status of these cells and their ability to differentiate into phagocytes *in vitro*. *In vivo*, *P. acnes* treatment elevated the absolute number of all B-1 subsets, whereas PS only increased B-1c. Moreover, the bacterium increased the number of B-1b cells that were positive for MHC II, TLR2, TLR4, TLR9, IL-4, IL-5 and IL-12, in addition to up-regulating TLR9, CD80 and CD86 expression. PS increased B-1b cell expression of TLR4, TLR9, CD40 and CD86, as well as IL-10 and IL-12 synthesis. Both of the treatments decreased the absolute number of B-1b cells *in vitro*, suggesting their early differentiation into B-1 cell-derived phagocytes (B-1CDP). We also observed a higher phagocytic activity from the phagocytes that were derived from B-1b cells after *P. acnes* and PS treatment. The adjuvant effect that *P. acnes* has on B-1 cells, mainly the B-1b subtype, reinforces the importance of B-1 cells in the innate and adaptive immune responses.

## Introduction

B lymphocytes can be divided in B-2 cells, which are abundant in the spleen, lymph nodes and peripheral blood of mice and continuously arise from bone marrow precursors, and B-1 cells, which arise from fetal and neonatal progenitors early in life [1], are enriched in mouse peritoneal and pleural cavities and can be distinguished by the expression of specific surface markers (CD5, CD11b and CD43) [2].

Peritoneal B-1 cells are comprised of 3 subsets: B-1a (CD11b^+^ CD5^+^) cells, B-1b (CD11b^+^ CD5^−^) cells and B-1c cells (CD11b^−^ CD5^+^) [2], [3], with the last subset recently being described as a precursor of the others [4].

The production of natural IgM antibodies [5] has been described as a function of B-1 cells, and B-1b cells have been found to have specific roles in autoimmunity [6], antigen tolerance [7] and the increase in murine melanoma cell metastatic behavior and malignancy [8], [9]. In addition, *Escherichia coli-*derived LPS is known to induce B-1 stimulation *in vivo* and *in vitro* [10], [11], and the antigen-presenting function of peritoneal cells stimulated with CpG is mainly attributed to B-1b cells [12]. Our laboratory has also demonstrated that a non-adherent B-1b cell enriched population can be obtained after culturing peritoneal cells for 5 days [13] and that these cells become adherent and differentiate into B-1 cell-derived phagocytes (B-1CDP) when they are re-cultivated for more than 5 days [14].

Therefore, B-1 lymphocytes are involved in essential immune mechanisms and can be important targets for immune modulation by biological adjuvants. However, it is noteworthy that the B-1 cell response to antigen or adjuvants is poorly understood. Our group recently described how killed *Propionibacterium acnes* (*P. acnes*) and a purified soluble polysaccharide (PS) extracted from the bacterial wall increase the absolute number of peritoneal B-1a cells [15].

*P. acnes*, a Gram-positive bacillus, is the major constituent of the normal human adult skin microflora [16]. In mice, killed *P. acnes* induces biological effects that modulate the innate and acquired immune responses, causing increased phagocytic [17], [18] and tumoricidal activities in macrophages [15], [19], [20], increased antibody responses [21], [22] and increased resistance to different pathogens [22]–[24].

Recently [18], [25] we characterized a purified soluble polysaccharide that can be extracted from the *P. acnes* cell wall (PS) with phenol and is the bacterial component involved in several of the *P. acnes* adjuvant effects. Similarly to the whole bacterium, PS increased nitric oxide (NO) and TNF-α synthesis [18] and tumoricidal and phagocytic activities in peritoneal macrophages [15].

An important effect of *P. acnes* and PS is their capacity to direct the immune response toward a Th1 or Th2 response, as demonstrated in a murine model of immediate hypersensitivity (Th2 response) to ovalbumin (OVA) [26]. In this model, a Th2 response was potentiated when *P. acnes* or PS were administered simultaneously with OVA, but this result changed to a typical Th1 response when the mice were treated before OVA sensitization [25], [27].

We also found that killed *P. acnes* or PS exert a potent adjuvant effect on *Trypanosoma cruzi* (*T. cruzi*) DNA immunization, enhancing the *T. cruzi*-specific Th1 immune response and significantly decreasing mouse parasitemia after challenge [22].

These results suggest that *P. acnes* and PS act directly on antigen-presenting cells (APC), which is in agreement with the increased number and activation status of bone marrow-derived dendritic cells obtained from *P. acnes*- or PS-treated mice [28].

The mechanisms by which *P. acnes* acts on APCs seem to involve toll-like receptors (TLRs). The *P. acnes* effects, including pro-inflammatory cytokine synthesis [29], were shown to be related to TLR2 and TLR9 activation [30], [31]. Jugeau *et al.* [32] also demonstrated that *P. acnes* induces TLR2 and TLR4 expression on keratinocytes. In addition to the TLRs, the bacterium can also modulate co-stimulatory and MHC II molecules *in vivo* [28].

These data indicate that *P. acnes* preferentially acts on innate immune cells; primarily macrophages and dendritic cells. In fact, most *P. acnes* effects, such as pathogen resistance and phagocytic and tumoricidal activities, are related to the modulation of macrophages, usually those obtained from the peritoneal cavity.

Thus, considering the fact that B-1 cells participate in the innate and adaptive immune responses and *P. acnes* is a powerful adjuvant, we investigated the modulatory effects that *P. acnes* and the PS compound exert on peritoneal B-1 cells.

## Materials and Methods

### Animals

Six to eight week-old female or male BALB/c mice were used in all of the experiments.

### Ethics statement

All of the experiments were conducted with institutional animal care. This study was approved by the University Ethics Committee (ID number: 1210/2004).

### Antigens

#### Heat-killed *P. acnes* suspension

The bacteria (obtained from Instituto Adolfo Lutz, São Paulo, S.P., Brazil) were cultured in anaerobic medium (Hemobac, Probac, São Paulo, S.P., Brazil) for 3 days at 37°C and washed by centrifugation. The pellet was re-suspended in 0.9% saline and subjected to continuous water vapor for 20 min at 120°C. The protein concentration of the suspension was determined by the Bradford method [33].

#### Purified soluble polysaccharide (PS)

The polysaccharide extraction and purification were performed as previously described [18], [25]. Briefly, 30 ml of heat-killed *P. acnes* (800 µg of protein/ml) were mixed with 30 ml of 90% phenol and 30 ml of distilled water and incubated for 10 min in a 70°C water bath, as described by the Palmer and Gerlough [34] protocol for polysaccharide extraction. After centrifugation at 2,000× *g* and 4°C, the water phase and the polysaccharide-enriched ring were collected. This step was repeated 3 times. Three volumes of ethanol were added to each volume of the mixture. After an overnight incubation at 4°C, the precipitate was obtained by centrifugation and designated as the soluble polysaccharide (PS). The carbohydrate concentration was determined by the Dubois method [35].

### Treatment protocol

The mice received a single intraperitoneal (ip) injection of either 140 µg heat-killed *P. acnes* (n = 20) or 25 µg PS (n = 20) in a 350 µl injection. The control group (n = 30) consisted of mice that were treated with sterile 0.9% saline under the same conditions. After 24 h, the peritoneal exudate cells (PEC) were collected and analyzed by flow cytometry (*in vivo*) or subjected to a B-1b enriched culture protocol for 5 days (*in vitro*).

### B-1b cell enriched culture

The B-1b lymphocytes were cultured using the protocol described by Almeida *et al.* [13]. The cells were obtained by washing the abdominal cavities (peritoneal exudate) of the mice with RPMI (Sigma, St. Louis, MO, USA). Then, 4×10^6^ cells/15 ml were cultured for 1 h at 37°C in a 5% CO_2_ atmosphere. After this time, the non-adherent cells were discarded. The adherent cells remained in the culture bottle, to which 15 ml of RPMI containing 10% heat-inactivated fetal bovine serum (R10) were added. The cells were incubated in the same conditions for 5 days without changing the culture medium. After this period, the non-adherent cells were collected and analyzed by flow cytometry.

It is important to note that this culture provides a B-1b enriched population, which means that, although B-1b lymphocytes are the major subset, other cell types can also be present. For this reason, we not only analyzed the number and activation status of the B-1b cells in these cultures but also the number and status of the B-1a and B-1c cells.

### Absolute number of B-1 cells

The cell suspensions obtained from the PEC or the cultures were both counted with Trypan Blue to determine the cellular viability and concentration (total absolute number of cells/ml). The cells were incubated with 5% normal mouse serum for 30 min at 4°C to block Fc receptors. The cells were washed and stained with FITC-conjugated anti-mouse CD23, PE-conjugated anti-mouse CD19, PerCP-conjugated anti-mouse CD5 and APC-conjugated anti-mouse CD11b (Pharmingen, San Diego, CA, USA) for 1 h at 4°C. All of the samples were fixed with 1% paraformaldehyde for 30 min at 4°C. The stained cells were evaluated using a FACSCalibur flow cytometer (Becton Dickinson, San Jose, CA, USA), and the B-1a, B-1b and B-1c populations were analyzed by CellQuest Pro software (Becton Dickinson, San Jose, CA, USA).

The B-1a and B-1b lymphocyte analyses were performed concomitantly in two gates. The first gate contained small cells corresponding to lymphocytes, whereas the second gate contained larger, more granular cells that generally correspond to macrophages. We evaluated the B-1 lymphocytes in the second gate because it has been recently demonstrated that B-1 cells, which also express CD11b^+^, form tightly associated doublets that are often present in the macrophage gate [4]. We considered the absolute numbers of B-1a and B-1b cells to be the sum of the values obtained in both gates.

The percentages were obtained based on 10,000 events. The absolute cell number for each treated group was calculated from the mean of the total cell number contained in 1 ml of PEC per mouse or the mean of the total cell number contained in 1 ml of culture.

### Co-stimulatory and MHC II molecule expression

The cells obtained from the PEC or the B-1b enriched cultures were analyzed for co-stimulatory and MHC II molecule expression by flow cytometry. To verify the expression of co-stimulatory and MHC II molecules in the lymphocyte population, the cells were stained with biotin-conjugated anti-mouse CD40, CD80 or CD86 followed by FITC-conjugated streptavidin or FITC-conjugated anti-mouse I-A^d^ (MHC II), PE-conjugated anti-mouse CD19, PerCP-conjugated anti-mouse CD5 and APC-conjugated anti-mouse CD11b (PharMingen). The cell percentages and mean fluorescence intensity (MFI) were obtained. The absolute cell number in each treatment group was calculated as described above.

### Extracellular TLR expression

The expression of TLR2, TLR4 and extracellular TLR9 was also analyzed in the cells from the PEC or cultures by flow cytometry. TLR9 is located in an intracellular compartment, but was recently shown to be expressed on the cell membrane of human peripheral blood B lymphocytes [36].

To verify TLR2 and TLR4 expression in the lymphocyte population, the cells were stained with purified rat anti-mouse TLR2 or TLR4 (R&D Systems, Minneapolis, MN, USA) followed by a FITC-conjugated anti-rat antibody, PE-conjugated anti-mouse CD19, PerCP-conjugated anti-mouse CD5 and APC-conjugated anti-mouse CD11b (PharMingen). To determine TLR9 expression, the lymphocytes were incubated with purified rabbit anti-mouse TLR9 (Zymed, San Francisco, CA, USA) followed by a PerCP-conjugated anti-rabbit antibody, FITC-conjugated anti-mouse CD5, PE-conjugated anti-mouse CD19 and APC-conjugated anti-mouse CD11b. The stained cells were analyzed, and the cell percentages and mean fluorescence intensity (MFI) were obtained. The absolute cell number was determined as described above.

### Intracellular TLR9 expression and cytokine synthesis

The cells obtained from the PEC or culture were stained as described above to determine the B-1 subsets. The cells were then fixed with 1% paraformaldehyde for 15 min at 4°C and permeabilized with 200 µl of 0.2% Triton-X-100 for 6 min at room temperature in the dark. The cells were washed and stained with anti-TLR9 as described above. For cytokine detection, the B-1 lymphocyte subsets were labeled with PE-conjugated anti-mouse IL-4, PE-conjugated anti-mouse IL-5, FITC-conjugated anti-mouse IL-10 or PE-conjugated anti-mouse IL-12 (Pharmingen). The stained cells were analyzed by flow cytometry, and the cell percentages and mean fluorescence intensity (MFI) were obtained. The absolute cell number in each treatment group was calculated as described above.

### B-1b cell-derived phagocytes (B-1CDP)

The B-1b cells were obtained from the different treatment groups using the protocol described in Almeida *et al.* [13]. After 5 days in culture, the supernatant containing the non-adherent cells was harvested, centrifuged and re-cultured in R10 medium.

For the microscopic analysis, 2.5×10^5^ cells/ml (1 ml) were dispensed onto cover glasses inserted into 24-well tissue culture plates. These cells were re-cultured for 1, 9, 24 and 120 hours. The cover glasses were washed twice with PBS, and the adherent cells were fixed with 0.5% glutaraldehyde, stained with Giemsa, and analyzed by optical microscopy. For the RNA extraction procedure, 1×10^6^ cells were re-cultured in plastic dishes and maintained in culture for 5 days.

### Reverse transcriptase-polymerase chain reaction (RT-PCR) analysis for hematopoietic transcription factors

Total RNA was extracted from the cells in each treatment group before and after re-culturing for differentiation into phagocytes using the TRIzol protocol specified by the manufacturer (Invitrogen, Carlsbad, CA, USA). The first-strand cDNA was synthesized with Super-Script III RNase H reverse transcriptase using an oligo (dT) primer (Invitrogen).

The reagents and thermocycling profiles that were used have previously been described by Popi *et al*. [14]. Briefly, the concentration of cDNA in the different samples was calibrated using GAPDH cDNA. The PCR protocol consisted of a denaturing step at 94°C for 2 min, 30 cycles of denaturing at 94°C for 30 sec, annealing at the primer-specific temperature for 30 sec, and primer extension at 72°C for 30 sec, and a final extension at 72°C for 10 min. The PCR products were resolved on agarose gels and visualized by ethidium bromide staining. The images were captured and quantified using a Kodak Digital Science Electrophoresis Documentation and Analysis System 120 (Eastman Kodak Co., Rochester, NY, USA).

### B-1CDP phagocytic function

The B-1b cells were re-cultured in 24-well culture plates containing a cover glass in each well at a concentration of 2.5×10^5^ cells/ml. After 24 or 120 hours, the non-adherent cells were discarded and a *Saccharomyces cerevisiae* cell suspension was added to the adherent population at a concentration of 5 yeasts per cell (determined on the basis of the number of B-1b cells at the beginning of the experiment (1.25×10^6^ yeasts/ml), and incubated for 1 h. The cover glasses were washed 3 times with PBS, fixed with 0.5% glutaraldehyde and stained with Giemsa. Phagocytosis was quantified with oil-immersion microscopy (1000×). Two hundred cells were evaluated by counting the number of internalized yeast cells. The endocytic index (EI) was determined using the following relationship: EI = (percentage of cells containing at least one yeast cell)×(average number of yeast cells per phagocytic cell).

### Statistical analysis

Student's *t*-test was used to determine significant differences between the control group and each treatment group, and *p* values smaller than 0.05 (*p*<0.05) were considered significant.

## Results

### *P. acnes* increases the absolute number of mouse peritoneal B-1 cells

**Figure 1:**
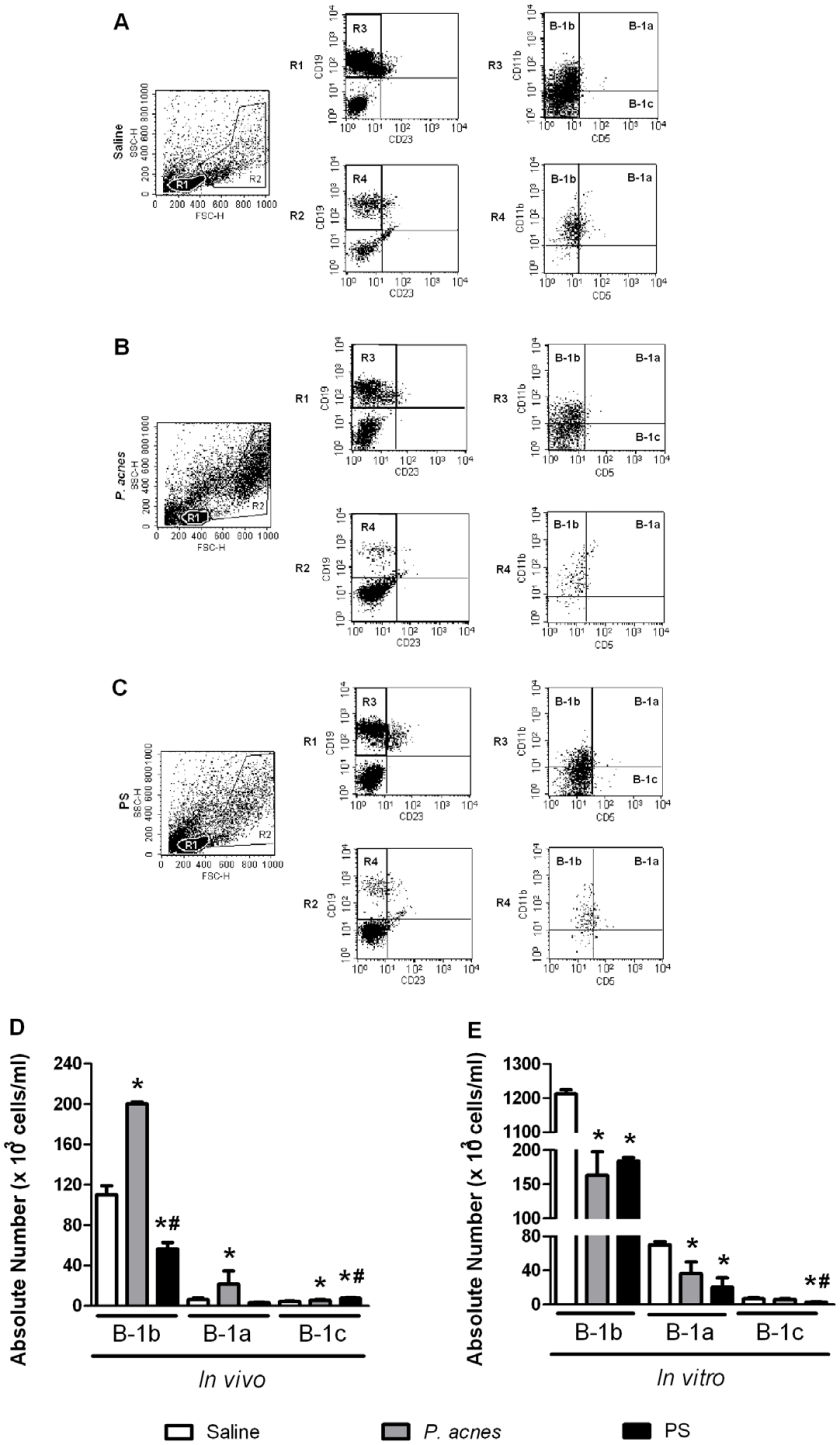
Determination of the absolute number of B-1 cell subsets *in vivo* and *in vitro*. Peritoneal cells from *P. acnes*-, PS- or saline-treated mice were analyzed 24 h after treatment (*in vivo*). The non-adherent population of 5-day-old B-1b enriched cultures was also analyzed (*in vitro*). The cells were stained with mAbs to identify the B-1 cell subsets. (A to C) The small (R1) and large granular (R2) cells from the SSC-H×FSC-H dot plots are shown, followed by the CD23×CD19 analysis from R1 and R2 and the CD5×CD11b analysis from R3 and R4. (D and E) The absolute numbers of B-1b, B-1a and B-1c lymphocytes from mice treated with saline (control group), *P. acnes* and PS. The absolute numbers of the B-1a and B-1b lymphocytes are the sum of R1 and R2, and the B-1c values are derived from R1. The absolute cell number is the mean of two independent experiments with similar results. * *p*<0.05 compared to the control group. ^#^ *p*<0.05 between the *P. acnes* and PS treated groups.

We first analyzed the B-1 cell subtypes obtained from the peritoneal cavity of BALB/c mice treated with saline (control group – Fig. 1 A), *P. acnes* (Fig. 1 B) or PS (Fig. 1 C). We analyzed the cells located in the R1 (small cells) and R2 (large granular cells) gates separately. All of the B-1 cells in both gates were CD19^+^ CD23^−^ (R3 and R4) (Fig. 1 A to C). Within the CD19^+^ CD23^−^ cell population, the B-1b subtype was characterized by a CD11b^+^ CD5^−^ phenotype (upper left quadrant in the R3 and R4 dot plots), whereas the B-1a cells were CD11b^+^ CD5^+^ (upper right quadrant in the R3 and R4 dot plots) and the B-1c cells were CD5^+^ CD11b^−^ (lower right quadrant in the R3 dot plot) (Fig. 1 A to C). We decided to include the data for the B-1a and B-1c cells in our statistical analysis (despite their low numbers) because the *P. acnes* and PS treatments exerted interesting effects on these small populations and their activation status, as described below.

Considering the sum of both the R1 (small cells) and R2 (doublets, large cells) gates, *P. acnes* treatment significantly enhanced (*p*<0.05) the absolute number of B-1b cells *in vivo* compared with the control group (Fig. 1 D). There was also a significant increase (*p*<0.05) in the absolute numbers of B-1a and B-1c cells in the *P. acnes*-treated mice compared with the control group, but these numbers remained lower than the absolute number of B-1b cells (Fig. 1 D).

In contrast to treatment with *P. acnes*, PS treatment decreased the absolute number of B-1b cells compared with the control treatment (Fig. 1 D), but significantly increased (*p*<0.05) the absolute number of B-1c cells compared with the saline- or *P. acnes*-treatments (Fig. 1 D).

### Impairment of B-1 subsets in a B-1b enriched cell culture from *P. acnes*- and PS-treated mice

The B-1b enriched cell cultures were analyzed by flow cytometry as described above (Fig. 1 A to C). All three B-1 subtypes were detected in the supernatant obtained from the 5^th^ day of *in vitro* culture, although B-1b remained the major population in all of the treatment groups (Fig. 1 E). The absolute number of B-1b cells was higher in the control group *in vitro* than *in vivo* (Fig. 1 E). This result was expected, because the enrichment of B-1b cells in culture has already been demonstrated [13]. The *P. acnes* and PS treatments significantly decreased (*p*<0.05) the absolute number of B-1b and B-1a cells present in the *in vitro* cultures compared with the control group (Fig. 1 E).

The absolute number of B-1c cells did not differ significantly between the *P. acnes* and control cultures. In contrast, the PS treatment significantly decreased (*p*<0.05) the absolute number of these lymphocytes compared with the saline and *P. acnes* treatments (Fig. 1 E).

### *P. acnes* and PS activated B-1 cell subsets, modulating cell surface molecule expression and cytokine synthesis *in vivo*

To analyze the molecular effects that *P. acnes* and PS have on peritoneal B-1 cell activation, we studied the expression of the TLRs, MHC II, CD40, CD80, CD86 and cytokines by these cells 24 h after treatment. Undoubtedly, the bacterium and the polysaccharide compound influenced the activation of the B-1b cells more than the other subsets, perhaps because this was the major B-1 subset in the mouse peritoneal cavity.

**Figure 2:**
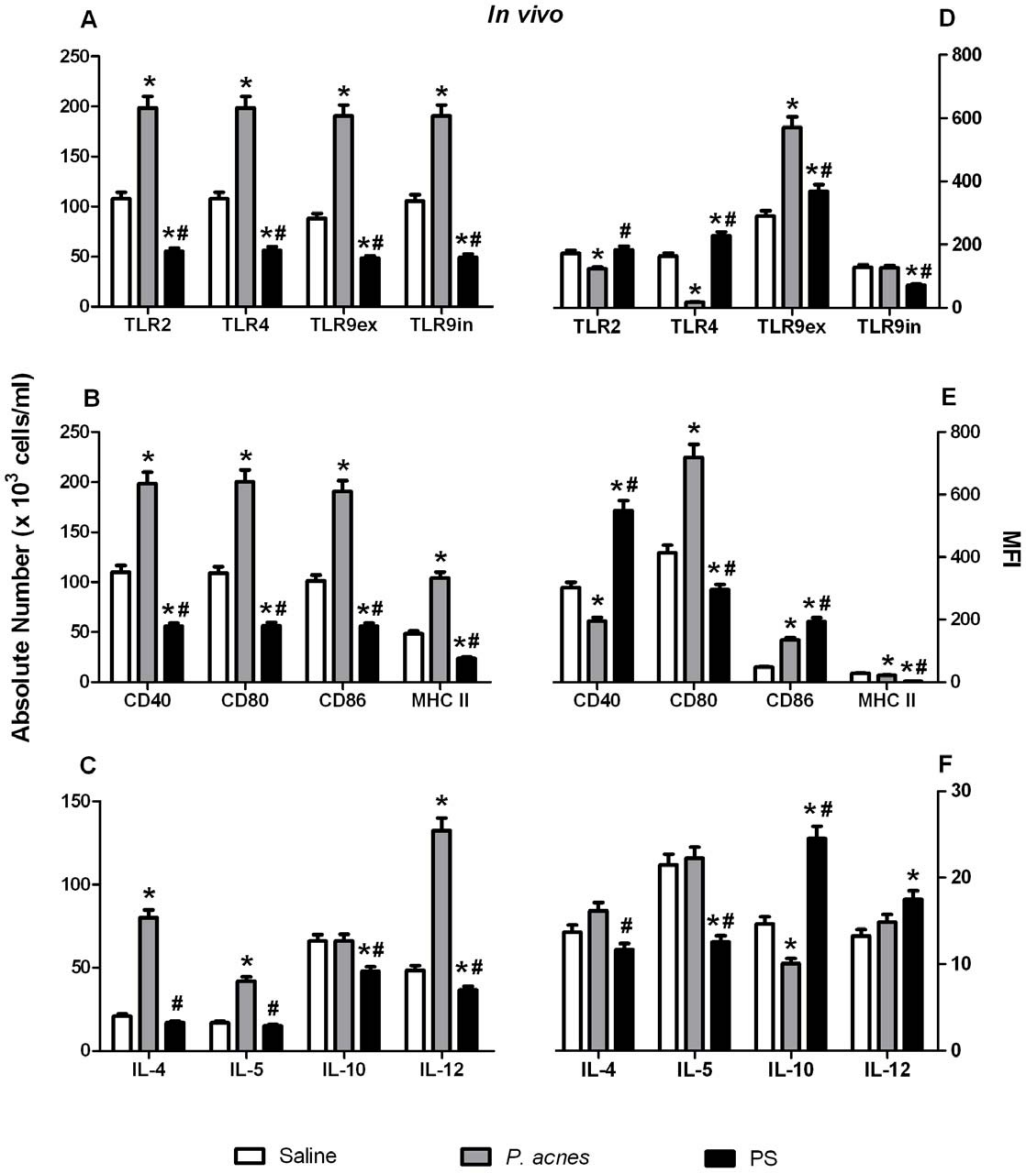
Analysis of the activation status of B-1b lymphocytes *in vivo*. Cells from *P. acnes*-, PS- or saline- (control group) treated mice were analyzed 24 h after treatment to determine TLR, co-stimulatory molecule, MHC II and cytokine expression by B-1b lymphocytes. The cells were stained with mAbs to determine the absolute number (A to C) of B-1b lymphocytes that expressed each molecule and the mean fluorescence intensity (MFI) of each marker (D to F). The absolute cell number and MFI are the means of two independent experiments with similar results.* *p*<0.05 between the control and treated groups. ^#^ *p*<0.05 between the *P. acnes* and PS treated groups.

*P. acnes* significantly increased the absolute number of B-1b cells expressing all of the toll-like receptors studied here (TLR2, TLR4, intra- and extracellular TLR9) and the absolute number of B-1b cells expressing MHC II and co-stimulatory molecules (Fig. 2 A and B). In contrast, PS decreased the absolute number of these cells compared to the saline and *P. acnes* treatments (Fig. 2 A and B). The bacterium also increased the absolute number of B-1b cells synthesizing IL-4, IL-5 and IL-12, whereas PS reduced the number of cells positive for IL-10 and IL-12 (Fig. 2 C).

When we determined the expression intensity (MFI) of these cell surface molecules, we observed that the *P. acnes* treatment caused the B-1b lymphocytes to significantly up-regulate their expression of extracellular TLR9, CD80 and CD86 relative to the control group and that PS caused these cells to up-regulate TLR4 and extracellular TLR9, as well as CD40 and CD86 (Fig. 2 D and E). *P. acnes* decreased the level of IL-10 produced by these cells, but PS significantly increased IL-10 and IL-12 synthesis (Fig. 2 F).

Similar to the results observed in the B-1b lymphocytes, *P. acnes* also increased the absolute number of B-1a cells that were positive for all of the TLRs, activation molecules and cytokines studied. PS diminished the absolute number of these cells compared with the control and *P. acnes* treatments (Fig. S1 A to C). The extracellular TLR9 levels, CD80 expression and IL-5 production were also up-regulated in the *P. acnes* group, and TLR2, TLR4, extracellular TLR9, CD86 and IL-12 were up-regulated in the B-1a cells from the PS-treated mice (Fig. S1 D to F).

In contrast, the *P. acnes* and PS effects on the B-1c cell activation molecules were slightly different from the effects these treatments exerted on the B-1b and B-1a cells, with the B-1c cells exhibiting an increase in the number of cells positive for TLR2, TLR4 and MHC II, but a decrease in the absolute number of cells expressing extracellular TLR9, CD40, CD80 and CD86 (Fig. S2 A and B). In addition, the *P. acnes* treatment also elevated the number of these cells synthesizing IL-4 and IL-5, whereas PS increased the number of IL-4, IL-10 and IL-12 positive cells (Fig. S2 C). Interestingly, *P. acnes* did not modulate the expression of any activation molecule on the B-1c lymphocytes, but increased the production of IL-5 and IL-12, whereas PS only up-regulated TLR4, extracellular TLR9, CD40 and CD86 expression (Fig. S2 D to F).

### B-1 cells from the *P. acnes*- and PS-treated mice remained activated in B-1b enriched cultures, despite a decrease in absolute number

Because the *P. acnes* and PS treatments modulated the levels of all the B-1 lymphocytes *in vivo*, and considering that all three subsets were detected in the B-1b enriched cultures, we decided to evaluate how these lymphocytes could be modulated by *P. acnes* and PS *in vitro*.

**Figure 3:**
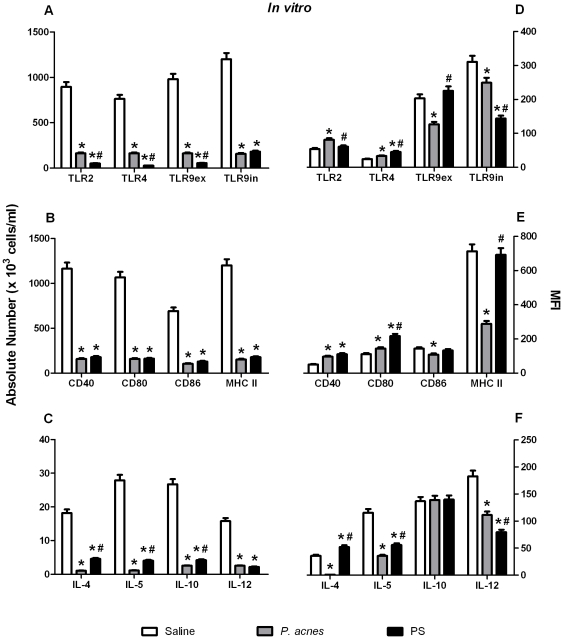
Analysis of the activation status of B-1b lymphocytes *in vitro*. The non-adherent cell population that was obtained after 5 days in a B-1b enriched culture obtained from *P. acnes*-, PS-, or saline- (control group) treated mice was analyzed to determine TLR, co-stimulatory molecule, MHC II and cytokine expression in B-1b lymphocytes. The cells were stained with mAbs to identify the absolute numbers (A to C) of B-1b lymphocytes that expressed each molecule and the mean fluorescence intensity (MFI) of each marker (D to F). The absolute cell number and MFI are the means of two independent experiments with similar results. * *p*<0.05 between the control and treated groups. ^#^ *p*<0.05 between the *P. acnes* and PS treated groups.

As described above, *in vivo* treatment with *P. acnes* or PS decreased the absolute number of B-1b and B-1a cells in the supernatant obtained from the 5^th^ day of culture. Consequently, the absolute number of B-1b and B-1a cells staining positively for the TLRs, MHC II, co-stimulatory molecules and cytokines *in vitro* also decreased in the *P. acnes* and PS groups compared with the control group (Fig. 3 and S3 A to C). The B-1c lymphocytes were an exception in this case because, although the absolute number of these cells decreased (mainly in the PS group), the number of cells positive for TLR2, extracellular TLR9 and MHC II increased in both treatment groups, as did the levels of IL-10 and IL-12 in the *P. acnes* group (Fig. S4 A to C).

Despite their lower cell numbers, the B-1b lymphocytes obtained from the *P. acnes* and PS peritoneal cell cultures remained more activated than the cells from the control group, as evidenced by higher TLR4, CD40 and CD80 expression in both treatment groups, higher TLR2 expression in the *P. acnes* group and higher IL-4 synthesis in the PS group (Fig. 3 D to F).

The B-1a cells from the treated groups also maintained their activation status *in vitro* despite their lower absolute number, as demonstrated by the up-regulation of extracellular TLR9 and CD80 in the *P. acnes* culture and the up-regulation of TLR2, TLR4 and extracellular TLR9 in the PS culture (Fig. S3 D and E). None of the treatments increased the cytokine synthesis by the B-1a lymphocytes *in vitro* (Fig. S3 F).

Finally, the B-1c lymphocytes from the *P. acnes* and PS cell cultures also remained more activated than the B-1c cells in the control culture, as evidenced by the up-regulation of extra- and intracellular TLR9, MHC II expression and IL-10 synthesis in the *P. acnes* treatment and the up-regulation of TLR2, extra- and intracellular TLR9, MHC II and IL-4 in the PS treatment (Fig. S4 D to F).

Although all of the B-1 subsets were detected in the supernatants from the 5^th^ day of culture and were activated by the *P. acnes* and PS treatments, it is unquestionable that the B-1b lymphocytes were the major population in these cultures and that *P. acnes* and PS exerted stronger effects on this subset. In addition, because this subpopulation has been shown to differentiate into phagocytes *in vitro*, we decided to investigate the effects that *P. acnes* and PS specifically have on the differentiation process.

### *P. acnes* induced the early *in vitro* differentiation of B-1b cells into phagocytes

As described above, when the non-adherent B-1b lymphocytes obtained from the 5 day old peritoneal cell culture are re-cultivated for an additional 5–10 days, they become adherent and differentiate into phagocytes [14]. The B-1b cells that were obtained from the saline-, *P. acnes*- or PS-treated groups of this study were re-cultivated for different periods (1 to 120 hours), and the phagocyte differentiation was analyzed by adherence and morphology.

**Figure 4:**
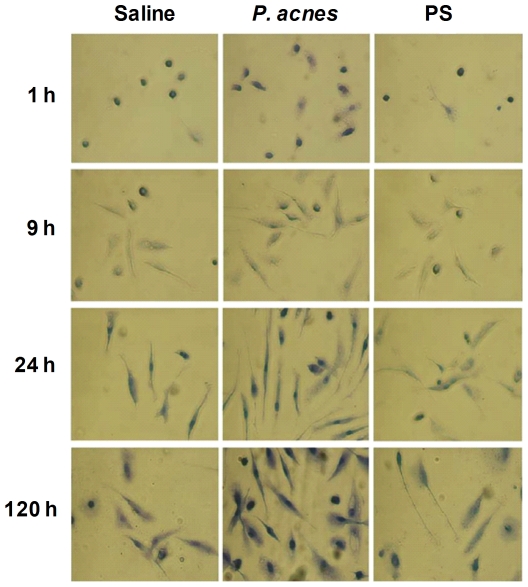
Differentiation of B-1b cells into B-1 cell-derived phagocytes (B-1CDP). The peritoneal cells from mice treated with *P. acnes*, PS or saline (control group) were cultured for 5 days. The non-adherent cells were re-cultured on cover glasses for 1 to 120 hours for phagocyte differentiation. The cells that adhered to the cover glasses were then fixed, stained with Giemsa and analyzed using optical microscopy. Magnification 500×.

We observed that the B-1b cells obtained from the *P. acnes* group differentiated into mononuclear phagocytes earlier, as there were more adherent lymphocytes in these cultures within the first hour and the cells were more spread out than those in the PS and control groups (Fig. 4).

There was an increase in the number of adherent cells in all three groups during the culture period, and these cells became large and projected pseudopods from two opposite cellular poles. These pseudopods became more prominent in the cells from the *P. acnes* group, and typical bipolar cells appeared within 9 to 24 hours (Fig. 4).

Although all three groups contained cells that were adherent and spread out at the end of re-culture period (120 hours), there were many more B-1 cell-derived phagocytes in the *P. acnes* cultures, whose cover glasses were almost completely filled (Fig. 4).

### The *P. acnes* and PS treatments induced the early myeloid commitment of B-1 cells, decreasing the expression of lymphoid genes

**Figure 5:**
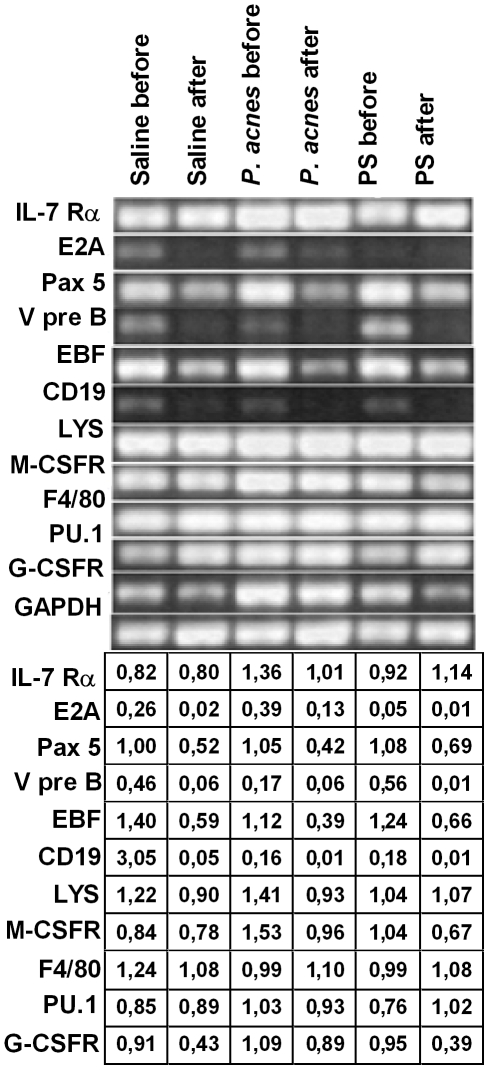
Myeloid and lymphoid gene expression by B-1b-enriched cells and B-1 cell-derived mononuclear phagocytes (B-1CDP). The peritoneal cells from *P. acnes*-, PS- or saline-treated mice were cultured for 5 days. The non-adherent population (B-1b-enriched cells) was re-cultured for 5 more days for phagocyte differentiation (B-1CDP). After each of these periods, the lymphoid and myeloid gene expression by B-1b cells (before re-culture) and B-1CDP (after re-culture) was analyzed by reverse transcriptase-polymerase chain reaction (RT-PCR). The PCR products were amplified using primers that detected the indicated transcripts. The PCR products were visualized on agarose gels by ethidium bromide staining. The amount of input cDNA was normalized by analyzing the control GAPDH transcripts. IL-7Rα: alpha subunit for interleukin 7 receptor, E2A: E box protein, Pax5: paired box 5, VpreB: surrogate light chain, EBF: early B-cell factor, LYS: Lysozyme; M-CSFR: macrophage colony-stimulating factor receptor, G-CSFR: granulocyte colony-stimulating factor receptor, GAPDH: glyceraldehyde 3-phosphate dehydrogenase.

The expression of lymphoid and myeloid genes was analyzed in B-1 cells obtained from the B-1b enriched population (before re-culture) and the B-1CDP cultures (after re-culture) (Fig. 5).

The cells from the B-1b enriched populations (before re-culture) that were obtained from the *P. acnes* and PS groups expressed lower levels of lymphoid genes such as CD19, VpreB and EBF (*P. acnes* group) and E2A (PS group) compared with the control group (Fig. 5). It is important to note that while the *P. acnes* and PS treatments decreased lymphoid gene expression in the B-1 cells, they sustained or increased the expression of myeloid genes such as lysozyme (Lys), M-CSFR, F4/80, PU.1 and G-CSFR (Fig. 5), indicating the early myeloid commitment of the B-1 cells from these groups.

When the B-1 lymphocytes were subjected to *in vitro* phagocyte differentiation (after re-culture), virtually all of the lymphoid genes were down-regulated in the cells from all three groups (Fig. 5). The expression of some myeloid factors also decreased after phagocyte differentiation, but genes such as F4/80 and PU.1, M-CSFR (saline group), G-CSFR (*P. acnes* group) and lysozyme (PS-treated group) remained sustained or increased (Fig. 5). The *P. acnes* and PS treatments clearly enhanced the myeloid commitment of the B-1CDP cells, as the down-regulation of some lymphoid genes was more pronounced in the treated groups after re-culture, with lower levels of Pax 5, EBF and CD19 in the *P. acnes* cultures and lower E2A, VpreB and CD19 levels in the PS cultures compared with the control cultures (Fig. 5). *P. acnes* also increased the expression of M-CSFR and G-CSFR in the B-1CDP cells relative to the control group (Fig. 5), further suggesting the enhanced myeloid commitment of these cells.

### *P. acnes* soluble polysaccharide increased the percentage of B-1CDP cells exhibiting phagocytic activity after 120 hours of re-culture

The B-1b enriched cells from the supernatant obtained on the 5^th^ day of culture were re-cultured for 24 or 120 hours to differentiate into phagocytes (B-1CDP). After each time period, *Saccharomyces cerevisiae* yeast cells were added to the cultures, and the phagocytic function of the cells was determined by calculating the phagocytic index.

**Figure 6:**
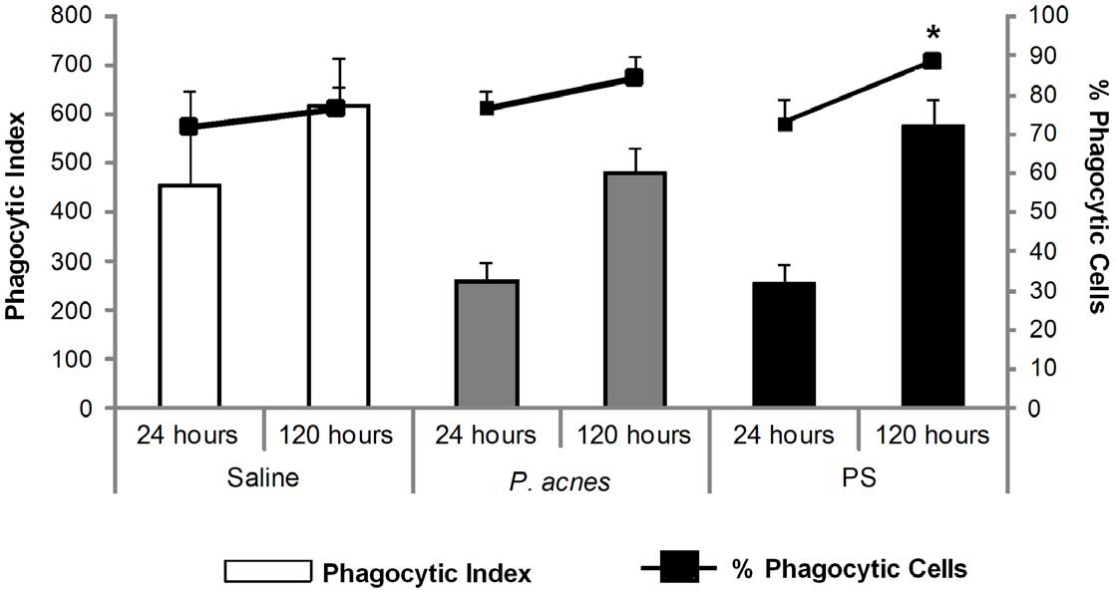
Evaluation of B-1CDP cell phagocytic activity. B-1b cells from the supernatant of 5 day-old cultures from the saline (control group), *P. acnes* and PS groups were re-cultured for 24 or 120 hours to differentiate into phagocytes (B-1CDP). *Saccharomyces cerevisiae* cells were added to the B-1CDP cells after each time point, and the phagocytic function was determined from the phagocytic index (bars) and the percentage of phagocytic cells (lines). * *p*<0.05 between the control and PS groups at 120 hours (percentage of phagocytic cells).

We did not observe significant differences in the phagocytic indices of the B-1CDP cells from the different groups at 24 or 120 hours after re-culture, but there was a tendency for the phagocytic index to be higher at 120 hours than 24 hours in all three groups (Fig. 6). There was no significant difference in the number of phagocytic cells obtained within 24 hours between the treated and control groups; however, the percentage of B-1CDP cells exhibiting phagocytic activity at 120 hours was significantly higher in the PS group than the control group (Fig. 6). Therefore, although *in vivo P. acnes* treatment enhanced the differentiation of B-1CDP cells *in vitro*, it did not enhance the cells' phagocytic function. Only the PS compound increased the number of B-1CDP cells exhibiting phagocytic activity.

## Discussion

In the present study, we demonstrated that *P. acnes* exerts an adjuvant effect on B-1 lymphocytes obtained from mouse peritoneal exudate cells. We also demonstrated that the soluble polysaccharide (PS) extracted from the bacterial wall is the compound responsible for some of the effects of the killed *P. acnes* suspension.

The levels of all B-1 subsets were elevated in the peritoneal exudate after *P. acnes* treatment; however, the B-1b cells remained the major subset. The adjuvant effect on absolute cell number could be related to three conditions: (1) proliferation, (2) differentiation and (3) cellular migration, all of which involve cell activation. Undoubtedly, both PS and *P. acnes* were able to activate B-1 cells *in vivo* by inducing changes in the expression of surface molecules and cytokine synthesis.

The increase in the absolute number of cells expressing TLR2, TLR4, and intra- and extracellular TLR9 was more pronounced in the B-1b cells than the other subsets and may reflect their proliferation and/or differentiation. *P. acnes* is a Gram-positive bacillus that preferentially binds to the cell surface through TLR2 [30]. This property is responsible for the majority of its adjuvant effects. The bacteria in the present study could have bound to B-1c cells, which have been described as a precursor of B-1 lymphocytes [4], and induced both cell proliferation and differentiation into B-1a and B-1b cells, which could explain the increase in the absolute number of all subsets (Fig. 1).

It also seems clear that the soluble polysaccharide extracted from the bacterial wall is the component responsible for increasing the number of B-1c cells because PS treatment only increased the absolute number of B-1c cells (Fig. 1), including those expressing TLR2 and TLR4 (Fig. S2 A). This effect could be due to the precursor nature of this subset, which would allow them to respond to a thymus-independent antigen [36] (such as PS) better than to the whole bacteria. *P. acnes* and PS have already been demonstrated to have effects on precursor cells that include increasing the absolute number of CD34^+^ bone marrow hematopoietic stem cells [28].

We also observed that *P. acnes* treatment differentially modulated TLR expression on the surface of B-1 lymphocytes, as determined by MFI. The whole bacterium only increased the extracellular TLR9 level. It has recently been shown that TLR9 is expressed on the cell membrane of human peripheral blood B lymphocytes [37]. Here, we are the first to demonstrate that B-1 lymphocytes from the mouse peritoneal cavity can also express extracellular TLR9 and that *P. acnes* modulates extracellular TLR9 expression. Despite failing to elevate the absolute number of B-1b cells expressing TLRs, PS did promote the higher expression of all TLR molecules (except intracellular TLR9) by this subtype (Fig. 2 D).

The polysaccharide-mediated interaction between bacteria and TLR2 could trigger other signals that result in differential TLR expression. In previous studies, TLR4 expression increased on hepatocytes from mice that were intravenously treated with *P. acnes* and challenged with LPS, demonstrating that the mechanism by which bacteria enhance endotoxic shock [38] is mediated by the up-regulation of TLR4 mRNA, which consequently enhances TLR4 expression on the cell surface [39]. An increase in the expression of both TLR2 and TLR4 was also demonstrated in keratinocytes and associated with the initial inflammatory process observed in acne lesions [32].

The bacterial-meditated up- or down-regulation of TLR expression could be directly related to B-1 lymphocyte traffic. Ha *et al.* [40] observed that peritoneal B-1 cell migration was regulated by TLR signals that induce the specific, rapid and transient down-regulation of integrins and CD9 on these cells, promoting their detachment from the local matrix and movement in response to chemokines. *P. acnes-*induced cell migration has recently been analyzed by our group. We observed higher numbers of dendritic cells, macrophages and NKT cells in the peritoneal cavity [15], [41]. Zhang *et al.* [42] demonstrated that bacterial treatment increased the synthesis of MIP-1α, a chemokine involved in monocyte and NKT recruitment, by rabbit macrophages.

TLR modulation by *P. acnes* could directly interfere with MHC II and co-stimulatory molecule expression and cytokine production by B-1 lymphocytes, especially the B-1b subtype. The number of B-1b cells expressing CD40, CD80, CD86, and MHC II and producing IL-4 and IL-12 increased after *P. acnes* treatment. This treatment also elevated CD80 and CD86 expression on the B-1b membrane. These results clearly show that the adjuvant effect of *P. acnes* influences B-1 cell antigen-presenting function. B-1b lymphocytes obtained from adherent peritoneal cell cultures have been demonstrated to be better antigen-presenting cells (APC) than macrophages *in vitro* [12]. B-1b cells have recently been shown to present internalized antigen to CD4^+^ T lymphocytes [43].

In contrast, several studies have described the adjuvant effect that *P. acnes* has on antigen-presenting cells such as dendritic cells, macrophages and B-2 lymphocytes [22], [28], [44], [45]. We observed a direct bacterial effect on APCs in a murine asthma model, with the Th2 response to OVA being potentiated or modulated depending on the *P. acnes* treatment protocol [25], [27].

The bacterial and PS-mediated modulation of B-1 lymphocytes *in vivo* led us to investigate how these cells would behave in the B-1b enriched culture that was described by Almeida *et al.* [13]. In these cultures, B-1b cells can be obtained from the supernatant of 5 day old adherent peritoneal cell cultures.

*P. acnes* and PS diminished the number of B-1b cells in the supernatant; however, these cells remained more activated than the cells from the saline-enriched cultures. After the B-1b lymphocytes obtained from the B-1b enriched cultures are re-cultured for 120 hours, a large proportion of them are found to be firmly adhered to the cover glass [13], [14]. These adherent cells spread to transform into B-1b cell-derived phagocytes (B-1CDP), which have a morphology that is similar to macrophages. We also demonstrated that B-1CDP cells express myeloid genes and silence some lymphoid genes at this stage [14].

In the present work, we observed that *P. acnes* transformed B-1b into B-1CDP cells at an early time point. When the B-1b enriched cultures from *P. acnes*-treated mice were re-cultured, they presented the characteristics of phagocytes within the first hour, as evidenced by the large number of adherent and spread cells with a bipolar shape (Fig. 4). Conversely, the cells in the other groups were small and round at the same time point (Fig. 4). *P. acnes* seems to anticipate the activation of intracellular pathways, silencing lymphoid genes and resulting in the characteristics observed in the B-1CDP cells.

Because Popi *et al.* [14] showed a relevant decrease in the expression of lymphoid genes after B-1b re-culturing, we analyzed the expression of lymphoid and myeloid genes in the B-1b and B-1CDP populations obtained from the *P. acnes*- and PS-treated groups.

*In vivo* treatment with *P. acnes* or PS silenced the lymphoid gene program in B-1CDP cells. The E2A and EBF transcription factors, which are involved in the early stages of B cell commitment, and Pax-5, which is a gene induced by E2A and EBF, were particularly affected. This finding clearly indicates that *P. acnes* or PS treatment induces myeloid commitment in B-1 cells and supports their differentiation into phagocytes after the lymphoid program is silenced and myeloid gene transcription is activated.

Almeida *et al*. [13] have already demonstrated the phagocytic activity of B-1b and B-1CDP cells *in vitro*. Thus, we evaluated whether *P. acnes* and PS could enhance such phenomena. We analyzed B-1CDP phagocytic function 24 and 120 hours after re-culture. Although the bacterium anticipated phagocyte differentiation, it did not potentiate the phagocytic function of the B-1CDP cells. It seems that the bacterium can influence the early differentiation of B-1b cells into phagocytes and enhance their APC characteristics.

In this study we have demonstrated that *P. acnes* treatment *in vivo* elevated the absolute number of B-1 cells and increased the expression of the TLRs, MHC II and co-stimulatory molecules, all of which are involved in capturing and processing antigens. These effects remained when the cells were cultured *in vitro*.

The adjuvant effect that *P. acnes* exerts on B-1 cell subtypes underscores the importance of B-1 cells in the early stages of the immune response.

## Supporting Information

Figure S1 **Analysis of the activation status of B-1a lymphocytes** ***in vivo*** **.** Cells from the *P. acnes*-, PS- or saline- (control group) treated mice were analyzed 24 h after treatment to determine TLR, co-stimulatory molecule, MHC II and cytokine expression by B-1a lymphocytes. The cells were stained with mAbs to determine the absolute number (A to C) of B-1a lymphocytes that expressed the studied molecules and the mean fluorescence intensity (MFI) of each marker (D to F). The absolute cell number and MFI are the means of two independent experiments with similar results.* *p*<0.05 between the control and treated groups. ^#^ *p*<0.05 between the *P. acnes* and PS treated groups.
(TIF)

Figure S2 **Analysis of the activation status of B-1c lymphocytes** ***in vivo*** **.** Cells from the *P. acnes*-, PS- or saline- (control group) treated mice were analyzed 24 h after treatment to determine TLR, co-stimulatory molecule, MHC II and cytokine expression by B-1c lymphocytes. The cells were stained with mAbs to determine the absolute number (A to C) of B-1c lymphocytes expressing the studied molecules and the mean fluorescence intensity (MFI) of each marker (D to F). The absolute cell number and MFI are the means of two independent experiments with similar results.* *p*<0.05 between the control and treated groups. ^#^ *p*<0.05 between the *P. acnes* and PS treated groups.
(TIF)

Figure S3 **Analysis of the activation status of B-1a lymphocytes** ***in vitro*** **.** The non-adherent cell population from the *P. acnes*-, PS-, or saline- (control group) treated mice was analyzed after 5 days in culture to determine TLR, co-stimulatory molecule, MHC II and cytokine expression by B-1a lymphocytes. The cells were stained with mAbs to determine the absolute number (A to C) of B-1a lymphocytes expressing the studied molecules and the mean fluorescence intensity (MFI) of each marker (D to F). The absolute cell number and MFI are the means of two independent experiments with similar results. * *p*<0.05 between the control and treated groups. ^#^ *p*<0.05 between the *P. acnes* and PS treated groups.
(TIF)

Figure S4 **Analysis of the activation status of B-1c lymphocytes** ***in vitro*** **.** The non-adherent cell population from the *P. acnes*-, PS-, or saline- (control group) treated mice was analyzed after 5 days in culture to determine TLR, co-stimulatory molecule, MHC II and cytokine expression by B-1c lymphocytes. The cells were stained with mAbs to determine the absolute number (A to C) of B-1c lymphocytes expressing the studied molecules and the mean fluorescence intensity (MFI) of each marker (D to F). The absolute cell number and MFI are the means of two independent experiments with similar results. * *p*<0.05 between the control and treated groups. ^#^ *p*<0.05 between the *P. acnes* and PS treated groups.
(TIF)
