# Supplementary figures and images for: Adjuvant Effect of Killed Propionibacterium acnes on Mouse Peritoneal B-1 Lymphocytes and Their Early Phagocyte Differentiation
Source: PLoS One. 2012 Mar 20;7(3):e33955. doi: 10.1371/journal.pone.0033955 (PMC3309018; doi:10.1371/journal.pone.0033955)

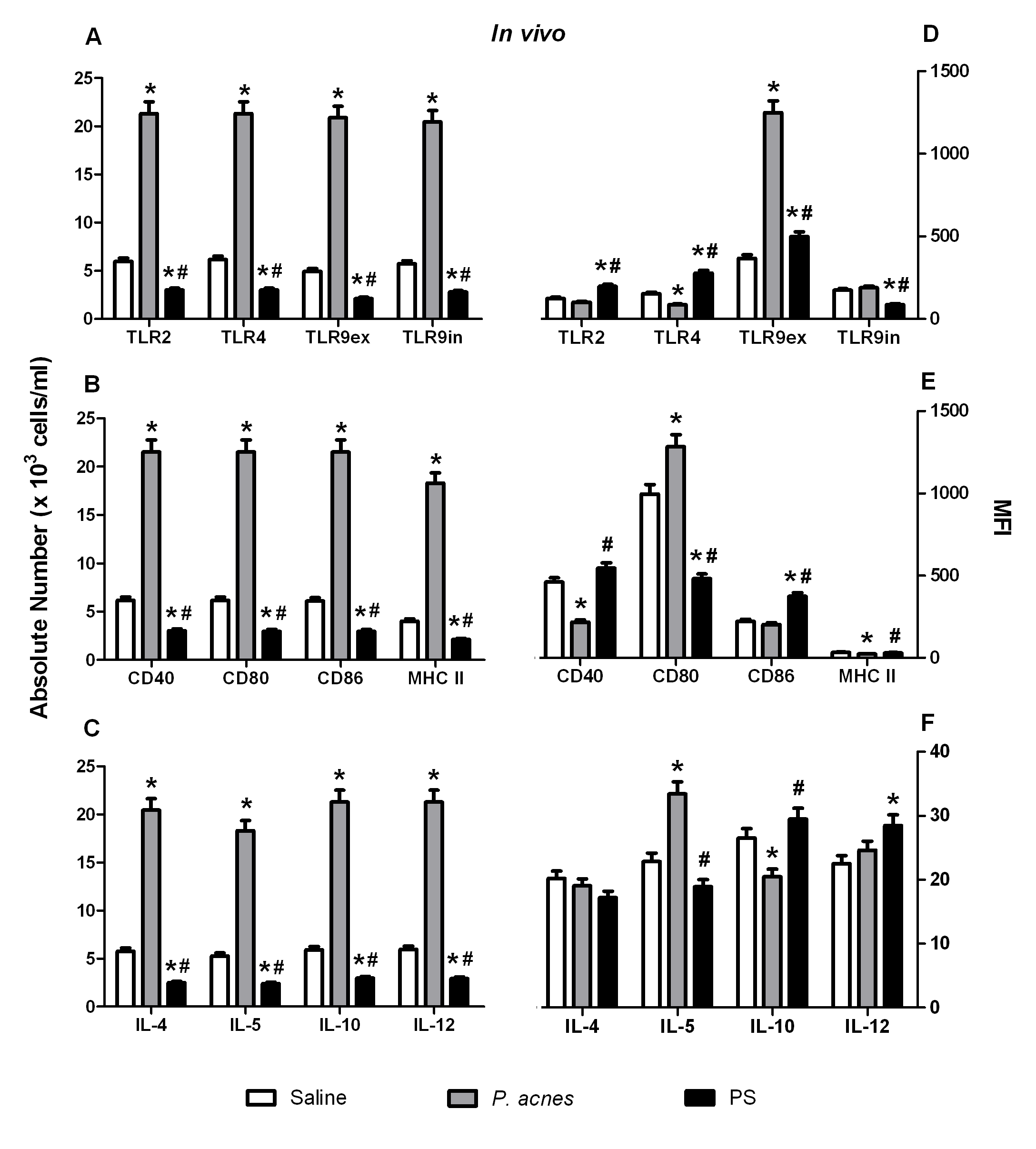

Supplement: Figure S1 — Analysis of the activation status of B-1a lymphocytes in vivo . Cells from the P. acnes-, PS- or saline- (control group) treated mice were analyzed 24 h after treatment to determine TLR, co-stimulatory molecule, MHC II and cytokine expression by B-1a lymphocytes. The cells were stained with mAbs to determine the absolute number (A to C) of B-1a lymphocytes that expressed the studied molecules and the mean fluorescence intensity (MFI) of each marker (D to F). The absolute cell number and MFI are the means of two independent experiments with similar results.* p<0.05 between the control and treated groups. # p<0.05 between the P. acnes and PS treated groups. (TIF) [file pone.0033955.s001.tif]

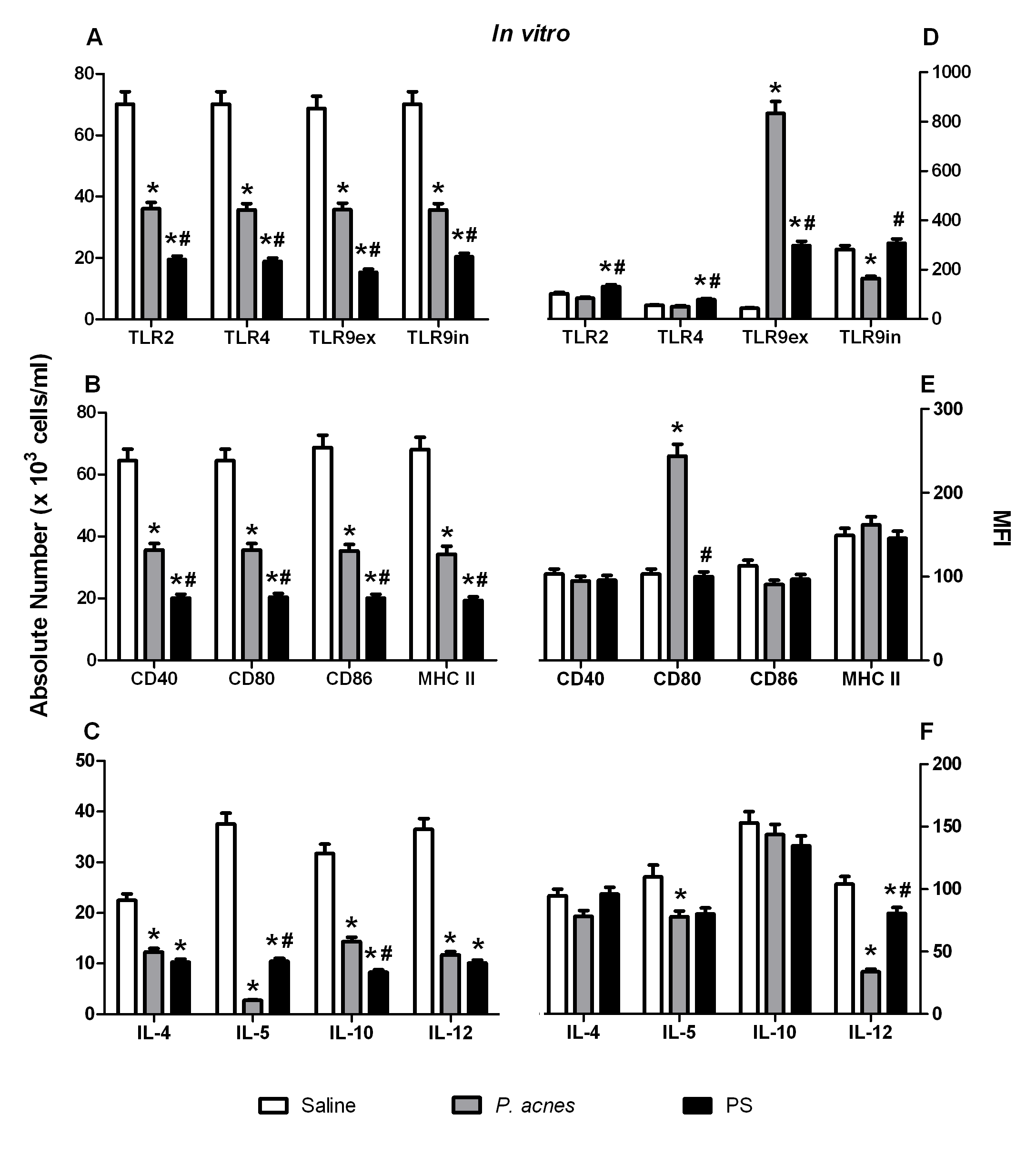

Supplement: Figure S2 — Analysis of the activation status of B-1c lymphocytes in vivo . Cells from the P. acnes-, PS- or saline- (control group) treated mice were analyzed 24 h after treatment to determine TLR, co-stimulatory molecule, MHC II and cytokine expression by B-1c lymphocytes. The cells were stained with mAbs to determine the absolute number (A to C) of B-1c lymphocytes expressing the studied molecules and the mean fluorescence intensity (MFI) of each marker (D to F). The absolute cell number and MFI are the means of two independent experiments with similar results.* p<0.05 between the control and treated groups. # p<0.05 between the P. acnes and PS treated groups. (TIF) [file pone.0033955.s002.tif]

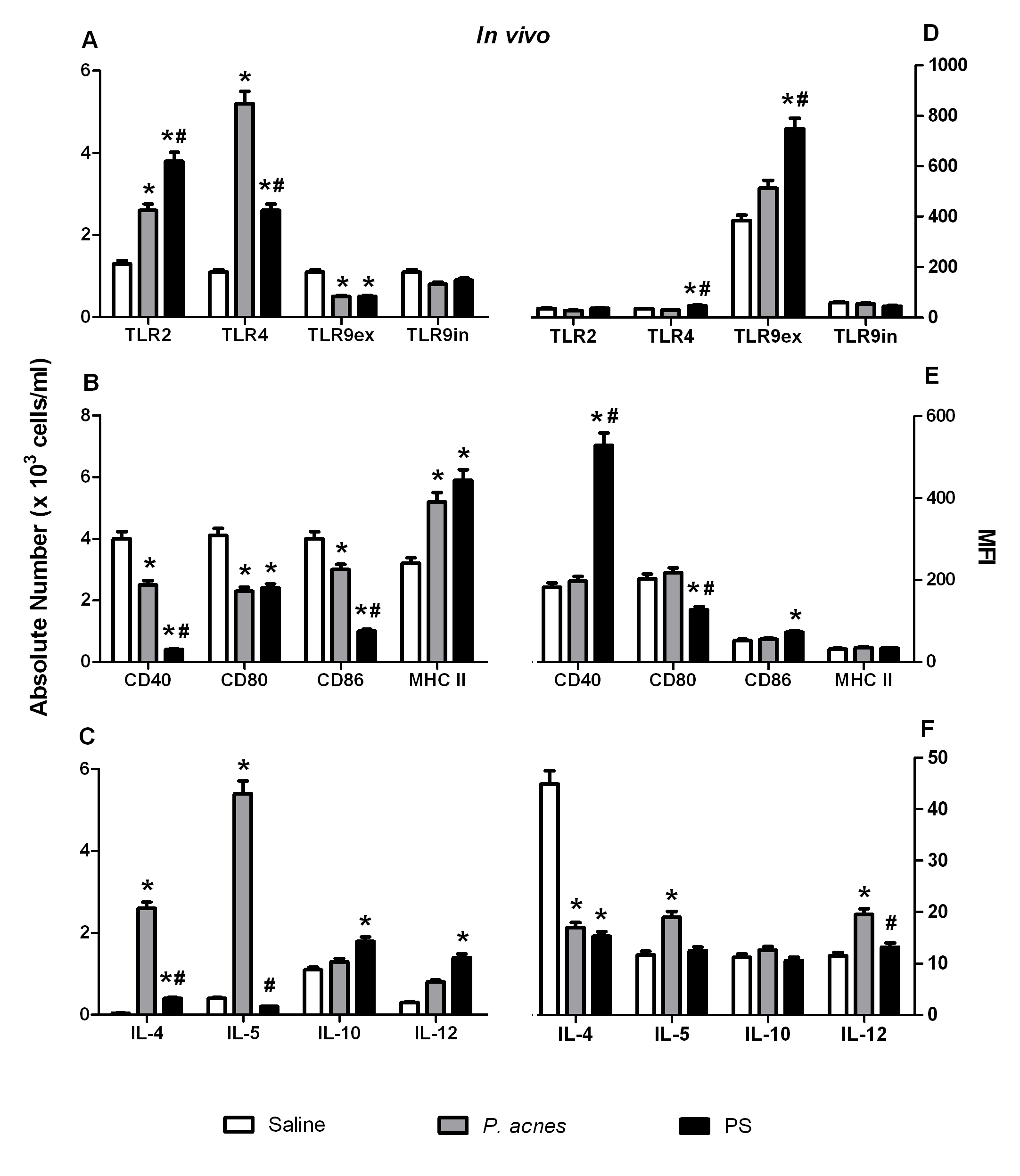

Supplement: Figure S3 — Analysis of the activation status of B-1a lymphocytes in vitro . The non-adherent cell population from the P. acnes-, PS-, or saline- (control group) treated mice was analyzed after 5 days in culture to determine TLR, co-stimulatory molecule, MHC II and cytokine expression by B-1a lymphocytes. The cells were stained with mAbs to determine the absolute number (A to C) of B-1a lymphocytes expressing the studied molecules and the mean fluorescence intensity (MFI) of each marker (D to F). The absolute cell number and MFI are the means of two independent experiments with similar results. * p<0.05 between the control and treated groups. # p<0.05 between the P. acnes and PS treated groups. (TIF) [file pone.0033955.s003.tif]

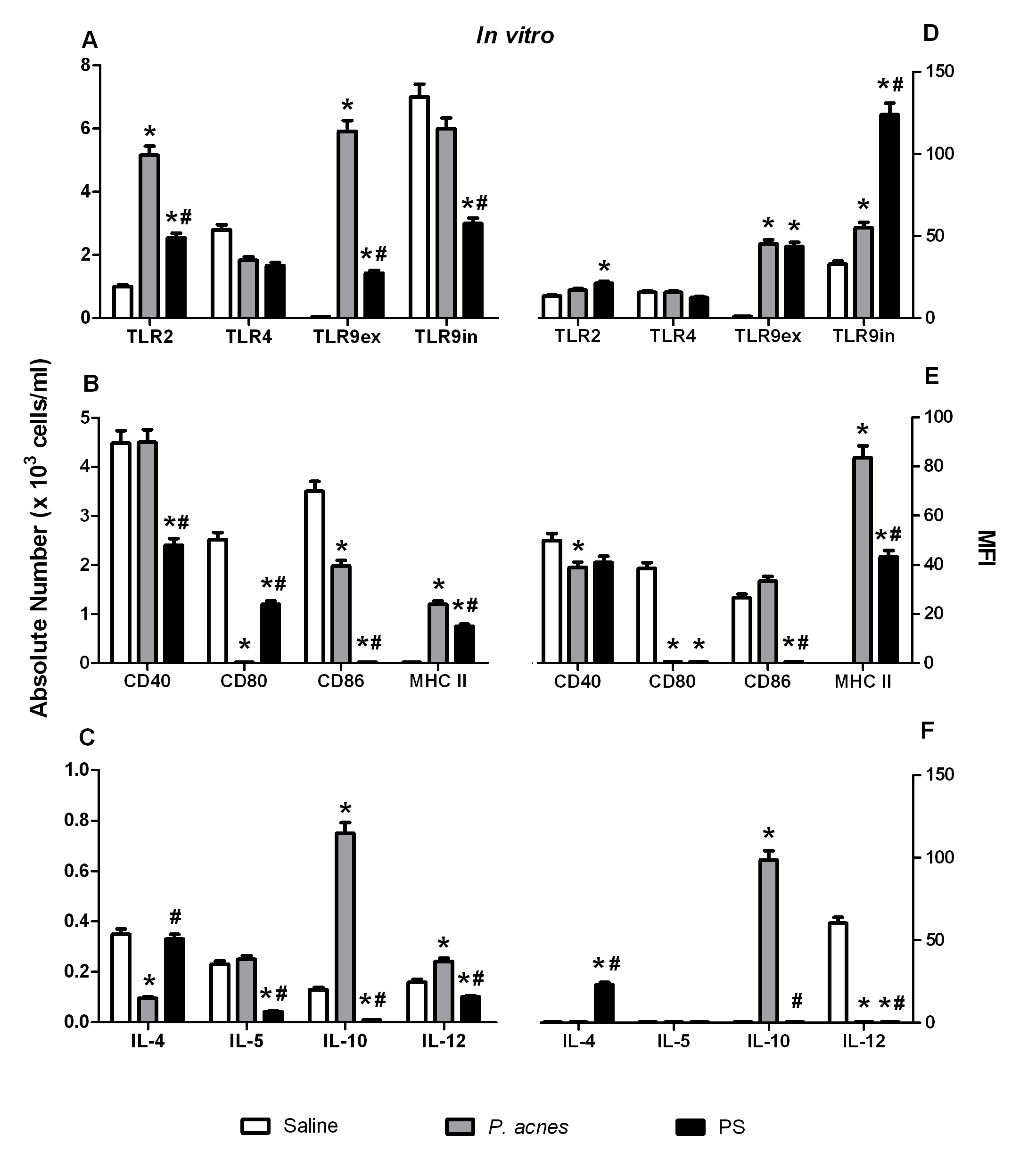

Supplement: Figure S4 — Analysis of the activation status of B-1c lymphocytes in vitro . The non-adherent cell population from the P. acnes-, PS-, or saline- (control group) treated mice was analyzed after 5 days in culture to determine TLR, co-stimulatory molecule, MHC II and cytokine expression by B-1c lymphocytes. The cells were stained with mAbs to determine the absolute number (A to C) of B-1c lymphocytes expressing the studied molecules and the mean fluorescence intensity (MFI) of each marker (D to F). The absolute cell number and MFI are the means of two independent experiments with similar results. * p<0.05 between the control and treated groups. # p<0.05 between the P. acnes and PS treated groups. (TIF) [file pone.0033955.s004.tif]
